# Experience With Medical Marijuana for Cancer Patients in the Palliative Setting

**DOI:** 10.7759/cureus.26406

**Published:** 2022-06-28

**Authors:** Karna T Sura, Leslie Kohman, Danning Huang, Silviu V Pasniciuc

**Affiliations:** 1 Department of Radiation Oncology, Upstate University Hospital, Syracuse, USA; 2 Department of Surgery, Upstate University Hospital, Syracuse, USA; 3 Department of Public Health and Preventive Medicine, Upstate University Hospital, Syracuse, USA; 4 Department of Internal Medicine, Upstate University Hospital, Syracuse, USA

**Keywords:** cancer, terminal care, quality of life, palliative care, medical marijuana

## Abstract

Objectives: Medical marijuana is a symptom treatment option for palliative cancer patients; however, its useful applications remain limited. The goals of this study were to review the characteristics of patients who received medical marijuana under our ambulatory palliative care program and to determine barriers to access and use of medical marijuana in this population.

Methods: This study was a retrospective analysis of patients who were enrolled in the medical marijuana registry through the ambulatory palliative care department at Upstate Cancer Center. Data from June 2017 to June 2020 were analyzed. Patients were included if they had a diagnosis of cancer, were certified by a qualified practitioner in the New York Medical Marijuana Program, and received care at Upstate Medical University. Patients were excluded if no marijuana certificate was found or if they transferred care.

Results: The study population was 184 patients. Ninety-three patients (51.5%) received at least one prescription from a New York licensed marijuana dispensary while 72 (39.13%) were certified but never obtained any medical marijuana. For patients who took at least one dose of medical marijuana, 48.14% experienced an improvement in pain, 44.95% used fewer opioids, and 85.11% had an improvement in at least one symptom. Adverse effects were low at 3.72%.

Conclusion: Medical marijuana has an important role in the palliation of symptoms in advanced cancers with few adverse effects. There are still many barriers to effective use. More prospective research is needed to optimize delivery and dosing.

## Introduction

Medical marijuana is a supportive agent for multiple indications, including HIV and cancer [1]. In patients with cancer, medical marijuana may improve nausea, vomiting, pain, and anti-tumor activity [2-4]. Few patient-centered clinical trials have been performed with medical marijuana since marijuana remains a schedule I drug in the United States, restricting any federally supported research or in federally-funded intuitions [5]. Even though the federal government puts limits on marijuana, some states have made it possible for people to get it for recreational or medical use.

New York State launched its medical marijuana program on January 7, 2016. The goal of the program is to provide access to medical marijuana for a variety of patients through certification from qualified practitioners who underwent additional training. Through the program, the provider certifies that the patient qualifies on the basis of one or more approved underlying conditions (cancer is one of them) and one or more qualifying symptoms (including pain, nausea, and loss of appetite). After certification, the patient must register with the state and receive a registration card allowing them to purchase marijuana at a state-approved dispensary.

The goals of this study were to review the characteristics of patients who received medical marijuana under our ambulatory cancer palliative care program and to determine barriers to access and use of medical marijuana in this population.

## Materials and methods

This study was a retrospective analysis of patients who underwent palliative care treatment at Upstate Cancer Center. The study was considered IRB exempt by the Upstate Medical University IRB on December 15, 2019 (Project 1512564-1).

Data from June 2017 to June 2020 were analyzed. Patients were included if they had a diagnosis of cancer, certified and registered in the New York Medical Marijuana Program, and received care at Upstate Medical University. Patients were excluded if no marijuana certificate was found or if they transferred care. A total of 184 patients were included in this analysis who were registered by a qualified practitioner.

Study data were collected and managed using Research Electronic Data Capture (REDCap) electronic data capture tools hosted at Upstate Medical University [6,7]. REDCap is a secure, web-based software platform designed to support data capture for research studies, providing (1) an intuitive interface for validated data capture, (2) audit trails for tracking data manipulation and export procedures, (3) automated export procedures for seamless data downloads to common statistical packages, and (4) procedures for data integration and interoperability with external sources.

Medical marijuana products dispensed were collected from the New York (NY) prescription registry via the Prescription Monitoring Program. The estimated annual gross income (AGI) of each patient was calculated by zip code using a dataset compiled by incomebyzipcode.com. The cost of the patient’s purchase of medical marijuana was estimated from the dispensers’ website. The patient’s vital status information was recorded until the end of the study.

A series of comparisons between groups were performed using two-sample t-tests, Pearson’s chi-squared tests, or Fisher’s exact tests if more than 50% of expected cell counts were less than five. A p-value of <0.05 was considered statistically significant. Receiver operating characteristic (ROC) analysis was used for analyzing the optimal dosages and calculating sensitivity and specificity. The data analysis for this paper was performed using SAS© 9.4 (Cary, NC: SAS System) or R package pROC (1.16.2).

## Results

A total of 184 patients were included in this analysis. The median age was 60 years (range: 21-92 years). Table 1 demonstrates the patient characteristics. Ninety-three patients (51.5%) purchased medical marijuana at least once, while 72 (39.13%) were certified but did not purchase any. At the end of the study period, 98 (53.26%) patients were alive.

**Table 1:** Patient characteristics of the study population presented as number and percent.

Category	Subcategory	N (%)
Race	White	166 (90.22%)
	Hispanic or Latino	1 (0.54%)
	Black or African American	13 (7.07%)
	Asian or Pacific Islander	4 (2.17%)
Gender	Male	84 (45.65%)
	Female	100 (54.35%)
Insurance	Government	118 (64.13%)
	Private	62(33.70%)
	Uninsured	4 (2.17%)
Smoker	Yes	135 (73.37%)
	No	49 (26.63%)
Prior drug use	Yes	49 (26.63%)
	No	135 (73.37%)
Prior marijuana use	Yes	48 (97.96%)
	No	1 (2.04%)
Annual gross income (AGI)	≤$95,000	26 (16.05%)
	>$95,000 and ≤250,000	25 (15.43%)
	>$250,000 and ≤300,000	24 (14.81%)
	>$300,000 and ≤525,000	27 (16.67%)
	>$525,000 and ≤800,000	26 (16.05%)
	>$800,000	34 (20.99%)
Intent	Curative	42 (22.83%)
	Palliative	142 (77.17%)
Cancer type	Breast	25 (13.59%)
	Central nervous system	8 (4.35%)
	Lung	39 (21.20%)
	Head and neck	13 (7.07%)
	Gastrointestinal	46 (25%)
	Genitourinary	22 (11.96%)
	Gynecologic	2 (1.09%)
	Other	29 (15.76%)

For those 72 patients who did not use any medical marijuana, reasons included death (22%), unknown (19%), used own supply (15%), cost (15%), registration issue (14%), not needed (8%), dispensary issue such as dispensary couldn’t verify the medical marijuana card (3%), and other (4%). Table 2 shows the univariate analysis of factors associated with not obtained any medical marijuana. Significant associations include time between certification and death, type of cancer, and faster time to death. Table in the Appendices shows the univariate analysis of those using one prescription versus multiple prescriptions.

**Table 2:** Factors were associated with not using the medical marijuana program. *P-value <0.005. **P-value <0.05. AGI: annual gross income; CNS: central nervous system; H&N: head and neck; GI: gastrointestinal; GU: genitourinary; GYN: gynecology

Category	Subcategory	No	Yes	p-Value
Mortality	Alive	34 (47.22)	59 (63.44)	0.037*
	Dead	38 (52.78)	34 (36.56)	
Cancer type	Breast	9 (12.50)	13 (13.98)	0.041**
	CNS	3 (4.17)	3 (3.23)	
	Lung	17 (23.61)	20 (21.51)	
	H&N	3 (4.17)	8 (8.60)	
	GI	20 (27.78)	20 (21.51)	
	GU	6 (8.33)	14 (15.05)	
	GYN	1 (1.39)	1 (1.08)	
	Other	13 (18.06)	14 (15.05)	
Smoker	No	19 (26.39)	26 (27.96)	0.823
	Yes	53 (73.61)	67 (72.04)	
Gender	Male	36 (50.00)	40 (43.01)	0.372
	Female	36 (50.00)	53 (56.99)	
Insurance	Government	47 (65.28)	60 (64.52)	0.432**
	Private	25 (34.72)	31 (33.33)	
	Uninsured	0 (0.00)	2 (2.15)	
Intent	Curative	17 (23.61)	21 (22.58)	0.876
	Palliative	55 (76.39)	72 (77.42)	
Prior marijuana use	No	1 (4.55)	0 (0.00)	0.489**
	Yes	21 (95.45)	23 (100.00)	
Prior drug use	No	50 (69.44)	70 (75.27)	0.405
	Yes	22 (30.56)	23 (24.73)	
Race	White	62 (86.11)	85 (91.40)	0.481**
	Hispanic or Latino	0 (0.00)	1 (1.08)	
	Black or African American	8 (11.11)	5 (5.38)	
	Asian or Pacific Islander	2 (2.78)	2 (2.15)	
AGI	≤160,000	18 (25.35)	23 (25.27)	0.595
	>$160,000 and ≤320,000	19 (26.76)	21 (23.08)	
	>$320,000 and ≤650,000	20 (28.17)	21 (23.08)	
	>$650,000	14 (19.72)	26 (28.57)	
Age	n	72	93	0.232
	Mean (SD)	57.4 (12.95)	59.8 (12.73)	
	Median	57.5	61	
	Min, max	21, 81	31, 92	
Time to death (days)	n	38	34	0.021*
	Mean (SD)	134.0 (145.16)	215.7 (147.15)	
	Median	67.5	171	
	Min, max	2, 557	30, 511	

Ninety-three patients obtained a total of 417 products dispensed. Each of these 93 patients had a median of four products dispensed (mean: 6, range: 1-43). The patients refilled medical marijuana at a median of 25.5 days (mean: 56 days, range: 2-358). Table 3 is a summary of the products dispensed to all the patients. For patients who used medical marijuana during the study period, 48.14% experienced improvement in pain, 44.95% used fewer opioids, and 85.11% had an improvement in at least one symptom. Symptoms assessed include anxiety, nausea, pain, sleep, neuropathy, and appetite. A univariate analysis was completed to determine association between improved pain as well as decreasing narcotic use. The total milligrams (p = 0.0003 and p = 0.0002, respectively) and specific marijuana dispensary (p<0.001 and p<0.001, respectively) were significantly associated with both improved pain and decreasing narcotic use

**Table 3:** Summary of product use.

Category	Subcategory	N (%)
Product type	Capsule	77 (18.47%)
	Tincture	10 (2.40%)
	Oral spray	8 (1.92%)
	Vaporizer	221 (53.00%)
	Lotion	2 (0.48%)
	Powder	5 (1.20%)
	Lozenges	6 (1.44%)
	Chewable	8 (1.92%)
	Solution	80 (19.18%)
Ratio	100:1	1 (0.24%)
	150:0	3 (0.73%)
	15:0	1 (0.24%)
	15:1	5 (1.22%)
	1:1	97 (23.72%)
	1:100	61 (14.91%)
	1:2	5 (1.22%)
	1:20	200 (48.90%)
	1:50	30 (7.33%)
	20:1	1 (0.24%)
	2:1	5 (1.22%)
Total mg dispensed	<250 mg	109 (26.14%)
	250-400 mg	145 (37.77%)
	>400 mg	163 (39.09%)
Days supplied	<10 days	269 (64.51%)
	≥10 days	148 (35.49%)

**Figure 1:**
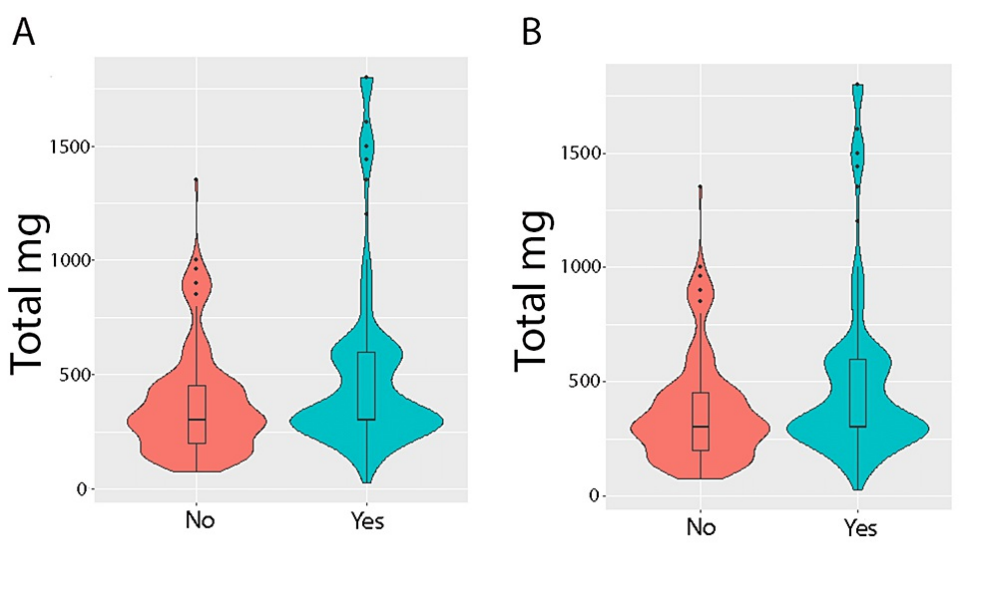
Plot of total milligrams versus (A) narcotic decreased and (B) pain improved

Figure 1 reviews the total marijuana dose per product with associations between narcotic use and pain improvement. Using ROC analysis, an optimal threshold was 470 mg per product purchased at one visit for both pain improvement and decreased narcotic use. For pain improvement, the sensitivity of the 470 mg dose was 0.374 (95% CI: 0.304-0.45) while the specificity was 0.818 (95% CI: 0.759-0.872). For narcotic use, the sensitivity of the 470 mg dose was 0.384 (95% CI: 0.308-0.459) while the specificity was 0.814 (95% CI: 0.759-0.864).

The reported adverse effects were low at 3.72%. The adverse effect breakdown included mild toxicity (five patients), financial (five patients), specific product intolerance (three patients), and severe toxicity (one patient). Mild toxicities included headaches, feeling bad, funny feelings, and dizziness. For the one patient with severe toxicity, the patient became drowsy and had a severe fall because of the drowsiness. The patient discontinued medical marijuana after this incident.

Financial aspects of medical marijuana were also reviewed. Fifty-five patients spent a median of $220 (mean: $625.90, range: $1.23-9020.50) on medical marijuana over the course of the data collection. Per month, patients spent a median of $59.28 per month (mean: $119.4/month, range: $0.68-604.92). If patients took the medication as recommended by the pharmacist, the cost would be a median of $294.83/month (mean: $893.10, range: $3.45-35417).

## Discussion

Our study reviewed the experience of oncology palliative care patients in our program with the New York Medical Marijuana Program. A majority of patients who were registered in the program purchased at least one product. For those who obtained any marijuana through the program, death at a short interval was the number one reason. Furthermore, death during the study period was associated with not using the program. There are many reasons for mortality to decrease the use of the program. One issue is the late referral for symptom relief and/or reluctance of the patient to try medical marijuana at end of life. Furthermore, it may be difficult to enroll and obtain medical marijuana at the end of life since there are multiple barriers [8]. New York State has a two-step process to obtain medical marijuana which requires certification and then the patient to fill out the form. Furthermore, medical marijuana needs to be obtained through a dispensary, which is limited in numbers and concentrated in big cities. Use of end of life, especially during hospice treatment, may be difficult due to need for social support who can obtain medical marijuana. More studies are necessary to understand barriers and medical marijuana use in hospice patients.

Many patients could not afford the high cost of medical marijuana, and it usually costs significantly more than “street” marijuana. Patients were spending a median of $60 per month in our study. However, if the patient were taking medical marijuana as prescribed by the pharmacist, the cost per month would be closer to $300 per month. Furthermore, we identified five patients who stopped treatment due to financial toxicity. Although we did not find an association between income and insurance type, we could not account for patients using medical marijuana sparingly because of the cost. Furthermore, there was no association with insurance type, but insurance companies do not cover any costs of medical marijuana currently. We did find that there was increased usage in the highest income group compared to the lowest income group. Insurance companies should make it a priority to cover these medications. Patients using their own supply like to be certified because they can support their recreational marijuana use medically in case of questioning by employers or law enforcement.

Medical marijuana improves at least one symptom in 85% of patients who took it. Pain improved and narcotic use decreased by 48% and 45%, respectively. These results are similar to findings by Schleider et al. that demonstrated an improvement in pain in 95.9% of patients [9]. Furthermore, patients may be using medical marijuana as a multi-symptom solution even outside cancer symptoms, for symptoms like anxiety and sleep disorders [10,11]. Medical marijuana’s improvement of pain and decrease in narcotic use may have a dose-dependent relationship. Our data demonstrated a dose-dependent relationship on improvement of symptoms, although this may be skewed by the higher doses. This dose dependency could also be affected by various forms of medical marijuana administration. Vinette et al. discussed the importance of tracking the multiple routes of administration for future studies because of the lack of information in the current literature [12]. Although medical marijuana dosage is not quantified through research, we found that optimal medical marijuana of 470 mg per product was beneficial. Further studies are necessary to validate dose dependency for symptoms, especially pain and narcotic use.

Another interesting relationship is associated with improvement in pain and narcotic use with certain dispensaries. In New York State, the practitioner doesn’t usually specify a product for medical marijuana. The pharmacist at the dispensary usually recommends the type and dose of medical marijuana for the patient based on prior history of marijuana use, symptom type and severity, and performance status. This association may speak to the importance of this consultation and different experiences of the pharmacist. Previous studies have demonstrated the important impact of pharmacists on educating patients with medical marijuana usage [10]. Of note, none of the dispensaries used by our patients provide smokable or edible forms of marijuana.

Adverse effects remain low at 4% in our study. Patients need to be informed of the dizzy, light-headed, and “funny” feeling of marijuana, which is similar to reported literature [13-16]. Only one patient had a severe adverse effect due to dizziness causing a fall. We did not observe any clinically symptomatic lung injuries in our cohort on medical marijuana, including those patients who were vaping.

Our study has severe limitations. This was a retrospective analysis at a single center. Dispensaries can change prices and selections, which could change the cost analysis. We could not verify that we captured every product the patient received since we had to rely on the NY prescription database, which only goes back one year. 

Our study adds to what is known about the nature of medical marijuana use among cancer patients. Medical marijuana does help cancer patients with a low risk of adverse effects. Prospective studies examining this treatment modality should be prioritized. A meta-analysis by Mücke et al. confirmed this need for further investigation [1]. Furthermore, questions remain about any anti-tumor properties that medical marijuana may possess although previous studies have demonstrated that over 50% of patients use marijuana for its anti-cancer properties [10]. Currently, we use medical marijuana for symptomatic support. The financial burden of medical marijuana could be quite high, especially if high therapeutic doses are needed for pain relief. There is quite a bit of enthusiasm in the palliative community to use medical marijuana; additional data will help clarify the risks and benefits of medical marijuana for cancer patients [17].

## Conclusions

Medical marijuana appears to have an important role in the palliation of symptoms in advanced cancers with few adverse effects although not all patients certified for use, go on to obtain it. There are many remaining barriers to effective use including financial toxicity and end-of-life care, introducing this so late in life that the benefit is limited. More prospective research is needed to optimize delivery and dose.

## Appendices

**Table 4:** Univariate analysis of those using one product versus multiple uses. *Fisher's exact test (used when < 5 individuals in a category). **P-value <0.05. AGI: annual gross income; CNS: central nervous system; H&N: head and neck; GI: gastrointestinal; GU: genitourinary; GYN: gynecology; N: no; Y: yes

Category	Subcategory		One prescription	More than one prescription	p-Value
AGI per 2017 IRS	2017 AGI ≤$95,000		1 (11.11)	10 (22.73)	0.147*
	2017 AGI >$95,000 and ≤250,000		0 (0.00)	10 (22.73)	
	2017 AGI >$250,000 and ≤300,000		0 (0.00)	5 (11.36)	
	2017 AGI >$300,000 and ≤525,000		3 (33.33)	5 (11.36)	
	2017 AGI >$525,000 and ≤800,000		1 (11.11)	6 (13.64)	
	2017 AGI >$800,000		4 (44.44)	8 (18.18)	
Mortality	Alive		6 (66.67)	37 (80.43)	0.392*
	Dead		3 (33.33)	9 (19.57)	
Cancer category	Breast		2 (22.22)	6 (13.04)	0.203*
	CNS		0 (0.00)	0 (0.00)	
	Lung		2 (22.22)	10 (21.74)	
	H&N		0 (0.00)	3 (6.52)	
	GI		0 (0.00)	11 (23.91)	
	GU		1 (11.11)	8 (17.39)	
	GYN		1 (11.11)	0 (0.00)	
	Other		3 (33.33)	8 (17.39)	
Smoker	N		6 (66.67)	10 (21.74)	0.013**
	Y		3 (33.33)	36 (78.26)	
Gender	Male		3 (33.33)	21 (45.65)	0.716*
	Female		6 (66.67)	25 (54.35)	
Insurance	Government		7 (77.78)	26 (56.52)	0.289*
	Private		2 (22.22)	20 (43.48)	
	Uninsured		0 (0.00)	0 (0.00)	
Intent	Curative		3 (33.33)	13 (28.26)	0.710*
	Palliative		6 (66.67)	33 (71.74)	
Marijuana use	N		0 (0.00)	0 (0.00)	0.710*
	Y		1 (100.00)	13 (100.00)	
Prior drug use	N		8 (88.89)	33 (71.74)	0.421*
	Y		1 (11.11)	13 (28.26)	
Race	White		7 (77.78)	43 (93.48)	0.184*
	Hispanic or Latino		0 (0.00)	0 (0.00)	
	Black or African American		1 (11.11)	2 (4.35)	
	Asian or Pacific Islander		1 (11.11)	1 (2.17)	
Reason	Used program	Yes	9 (100.00)	46 (100.00)	0.184*
		No	0 (0.00)	0 (0.00)	
		Unknown	0 (0.00)	0 (0.00)	
